# Association between whole-grain consumption, tryptophan metabolism and psychological distress: a secondary analysis of a randomised controlled trial

**DOI:** 10.1017/S0007114524001077

**Published:** 2024-08-14

**Authors:** Vilma Liikonen, Mari Näätänen, Anna Kårlund, Kati Hanhineva, Leila Karhunen, Marjukka Kolehmainen

**Affiliations:** 1 Department of Clinical Nutrition, Institute of Public Health and Clinical Nutrition, University of Eastern Finland, P.O. Box 1627, 70211 Kuopio, Finland; 2 Department of Biotechnology, Food Sciences unit, University of Turku, 20014 Turku, Finland

**Keywords:** Whole grains, Tryptophan metabolism, Psychological distress, Weight management

## Abstract

This study aimed to investigate whether psychological distress, whole-grain consumption and tryptophan metabolism are associated with participants undergoing weight management intervention. Seventy-nine women and men (mean age 49·7 (sd 9·0) years; BMI 34·2(sd 2·5) kg/m^2^) participated in a 7-week weight-loss (WL) period and in a 24-week weight maintenance (WM) intervention period. Whole-grain consumption was measured using 4 d food diaries. Psychological distress was assessed with the General Health Questionnaire-12 (GHQ), and participants were divided into three GHQ groups based on the GHQ scores before WL. Tryptophan metabolites were determined from the participants’ fasting plasma using liquid chromatography-MS. GHQ scores were not associated with the whole-grain consumption. A positive association was observed between the whole-grain consumption and indole propionic acid (IPA) during the WM (*P* = 0·033). Serotonin levels were higher after the WL in the lowest GHQ tertile (*P* = 0·033), while the level at the end of the WM was higher compared with other timepoints in the highest GHQ tertile (*P* = 0·015 and *P* = 0·001). This difference between groups was not statistically significant. Furthermore, levels of several tryptophan metabolites changed within the groups during the study. Tryptophan metabolism changed during the study in the whole study group, independently from the level of psychological distress. The association between whole-grain consumption and IPA is possibly explained by the effects of dietary fibre on gut microbiota. This broadens the understanding of the pathways behind the health benefits associated with the intake of whole grains.

Diet has an impact on health, and its importance in psychological well-being has been recently studied^(1)^. Psychological distress refers to versatile symptoms of stress, anxiety and depression^(2)^. In addition, higher levels of psychological distress are related to mental disorders, such as anxiety and depressive disorders^(3)^. A healthy diet including nutrient-dense and fibre-rich foods is associated with a reduced risk of depressive symptoms^(1,4)^. Nutrient-dense and fibre-rich foods include whole-grain products, whose health-promoting effects are possibly mediated by antioxidants, folate and fibre^(5)^. However, the causality between whole-grain-rich diet and mental health and the mechanisms underlying their associations are still unclear. On the other hand, psychological distress can affect food choices and thus the consumption of whole grains, indicating a reverse causation^(6,7)^. By understanding this association and acknowledging how these alterations in food choices may subsequently affect nutrient intake, it emphasises the significance of evaluating the association between psychological distress and food choices.

One of the potential mechanisms mediating the relationship between whole-grain consumption and mental health might be via tryptophan metabolism. Tryptophan is an essential amino acid that the human body cannot synthesise^(8)^. Therefore, the daily need must be covered by diet. It is present in whole grains in small concentrations, and tryptophan content varies from 125 to 169 mg/100 g, depending on the type of grain^(8,9)^. Moreover, the regulation of tryptophan metabolism is influenced by the gut microbiota and its metabolic processes^(10)^. The fibre present in whole grains can affect the composition and metabolic pathways of the intestinal microbiota and thus the tryptophan metabolism. In fact, we have previously shown that consuming cereal fibre led to changes in serum levels of gut-derived tryptophan metabolites, including serotonin^(11)^ and indole propionic acid (IPA)^(12)^. In addition to whole-grain and overall diet, weight is suggested to be related to tryptophan metabolism^(13,14)^. When attempting to lose weight, there may be changes in the levels of tryptophan and its metabolites^(15–17)^. Therefore, managing weight through dietary intervention, such as incorporating whole grains, could potentially play a role in regulating tryptophan metabolism and its associated health effects.

Tryptophan is a precursor for many neuroendocrine neurotransmitters affecting psychological well-being, such as serotonin^(18–20)^. Tryptophan can also be oxidised via the kynurenine and indole pathways, and metabolites from these pathways can cross the blood–brain barrier^(8,19,21,22)^. These tryptophan metabolites take part in regulation of emotions, cognition and other brain functions within the brain^(19,21)^. Nevertheless, the precise relationship between tryptophan metabolism and psychological distress is yet to be defined.

While a healthy diet rich in whole grains may alleviate symptoms associated with psychological distress, potentially through the mediation of tryptophan metabolism, and it is acknowledged that psychological distress can influence whole-grain consumption, there is still limited evidence regarding the associations between whole-grain consumption, tryptophan metabolism and psychological distress. Despite that a bidirectional relationship may exist between psychological distress and whole-grain consumption, this study focuses on assessing if the psychological distress is associated with whole-grain intake in this population. This study aimed to investigate (1) whether whole-grain consumption is related to plasma tryptophan metabolite levels, (2) whether tryptophan metabolite levels differ according to different levels of psychological distress and (3) whether psychological distress is associated with whole-grain consumption. We conducted a secondary analysis of our previous dietary weight management study and investigated the associations between whole-grain intake, psychological distress and tryptophan metabolism during the whole study and the weight maintenance (WM) period after the weight loss (WL)^(23)^. We hypothesised that (1) the use of whole grains is associated with beneficial pathways of tryptophan metabolism, (2) tryptophan metabolite levels differ according to psychological distress level and (3) higher psychological distress is related to lower whole-grain consumption.

## Materials and methods

### Participants

A total of ninety-nine participants with obesity started the intervention study. The intervention study was conducted according to the guidelines laid down in the Declaration of Helsinki, and all procedures involving human subjects were approved by the Ethics Committee of the District Hospital Region of Northern Savo (ethics no. 46/2008). The study was registered in isrctn.org with the identifier 67529475. Written informed consent was obtained from all subjects. Recruitment was made through advertisements in a local newspaper and among eligible individuals who had previously participated in the studies at the Institute of Public Health and Clinical Nutrition at the University of Eastern Finland. Inclusion criteria were BMI of 30–40 kg/m^2^ and age of 30–65 years. Exclusion criteria were BMI out of the inclusion range, type 1 or 2 diabetes, pregnancy, kidney or thyroid dysfunction, heart or liver disease, polycystic ovary syndrome, diagnosed eating disorder, alcohol consumption > 16 (women) or > 24 (men) portions/week (1 portion equals 12 g of pure alcohol), neuroleptic or oral cortisone medication or any other diseases or medications that could potentially affect the measurements or completion of the study^(23)^.

### Study design

This study is a secondary analysis of our previous randomised controlled trial^(23)^. Intervention protocol, selection of foods for intervention and randomisation protocol have been described in detail before^(23,24)^. In short, the participants took part in a 7-week WL period using very-low-energy-diet products (Nutrifast, Leiras Finland Ltd) providing 600 kcal/d. Low-energy vegetables and energy-free beverages were allowed to be consumed *ad libitum*. The WL was followed by a 2-week transition phase, after which the participants were stratified by age and sex and randomised into two diet groups for a 24-week WM period. During the WM, one group consumed diet containing higher-satiety intervention foods and the other isoenergetic lower-satiety intervention foods. This meant that the higher-satiety food group consumed more fibre-rich breads and protein-rich foods than the lower-satiety food group. The satiety values of the intervention foods were assessed beforehand in a separate test in controlled laboratory settings^(23)^. The intervention food products and nutrition values of intervention foods have been described in detail previously^(23,24)^. For each participant, the intervention foods covered about 30 % of the individual energy requirement calculated for the WM. Participants got written instructions indicating the daily portions of each test food to be consumed. Otherwise, participants were allowed to eat freely selected foods *ad libitum*. In the WM, participants were advised to maintain the WL achieved during the WL without actively reducing weight.

### Dietary intake

Whole-grain products were defined as cereal products containing ≥ 6 g/100 g of dietary fibre. Participants completed 4 d food diaries before WL (i.e. baseline) and at weeks 6, 12, 18 and 24 during the WM. The intake of cereal products containing≥ 6 g/100 g of dietary fibre (g/d) was calculated using Micro Nutrica® dietary analysis software version 2.5 (the Social Insurance Institution of Finland). Whole-grain consumption was calculated as the mean intake from food diaries collected during the WM, allowing for the consideration of normal variations in consumption between days.

Participants’ protein intake ranged from 49 to 143 g/d and fibre intake from 13 to 55 g/d during the WM, as assessed from the food diaries.

### Psychological distress

Psychological distress of the participants was assessed with the 12-item General Health Questionnaire (GHQ)^(25)^ before WL and at weeks 0, 12 and 24 during the WM. The shorter version used in this study has the advantage of brevity, and its validity is comparable to the longer, 28-item questionnaire^(26)^. GHQ-12 is designed to measure temporal psychological well-being by assessing symptoms of anxiety and depression, social dysfunction and loss of confidence^(27)^. Responses are given in a four-point Likert scale with higher scores indicating greater psychological distress.

To investigate the association between psychological distress and whole-grain consumption, the values obtained at week 24 of the WM were included in the analysis. The GHQ questionnaire evaluates symptoms over the past few weeks. Therefore, GHQ scores measured at the end of the WM reflect psychological distress during that period. To investigate the hypothesis regarding variations in tryptophan metabolites among participants with distinct levels of psychological distress, the participants were divided into three groups by manually splitting study group into tertiles based on the GHQ scores measured before the WL. The higher-satiety food and lower-satiety food intervention groups did not differ in the GHQ scores, as reported previously^(23)^, and thus, our analysis was focused on GHQ scores rather than intervention groups. Additionally, the intervention groups were not differently distributed into the three GHQ tertile groups (see Table 1).

**Table 1.** Characteristics of the participants in the GHQ tertile groups (mean values and standard deviations)

GHQ group	Lowest tertile		Middle tertile		Highest tertile		
	Mean	sd	Mean	sd	Mean	sd	*P*
GHQ scores before the WL*	6·4	1·3	9·9	0·9	16	3·7	<0·001
*n* †	24		27		27		0·923
Females/males	18/6		19/8		20/7		
Age (years)*	50·7	10·2	49·3	9·1	49·6	8·0	0·760
Body weight before the WL (kg)‡	95·1	10·8	96·6	11·8	94·7	13·6	0·836
BMI before the WL (kg/m^2^)‡	34·2	2·5	34·7	2·5	33·6	2·6	0·252
Weight change during the WL (kg)‡	–11·9	3·2	–13·1	3·2	–11·4	3·2	0·180
HSF/LSF†	11/13		13/14		16/11		0·583
Whole-grain consumption during the WM (g/d)‡	117·8	46·2	119·9	51·3	109·9	51·3	0·681
Fibre intake during the WM (g/d)‡	27·8	8·4	28·3	7·9	27·8	10·9	0·962
Energy intake during the WM (kcal/d)‡	1647	361	1798	396	1713	397	0·381
Protein intake during the WM (g/d)‡	89·8	22·0	95·3	23·3	92·4	23·8	0·693

GHQ, General Health Questionnaire; WL, weight loss; HSF, higher-satiety food; LSF, lower-satiety food; WM, weight maintenance.

The score ranges for the lowest, middle and highest tertiles are 3–8, 9–11 and 12–25, respectively.

* Groups are compared with the Kruskal–Wallis H test.

† Groups are compared with the χ^2^ test.

‡ Groups are compared with the one-way ANOVA.

### Anthropometric measurements

Weight was measured in the morning after a 12-hour fast before WL and at weeks 0, 12 and 24 of the WM^(23)^.

### Blood sampling and metabolomic analysis

Blood samples were collected during the study clinic visits before the WL and at weeks 0 and 24 of the WM. The samples were collected in the morning after a 12 h fast. Tryptophan metabolites were determined from fasting plasma samples using non-targeted liquid chromatography (LC) and MS-based (LC-MS) metabolomic approach^(28)^. The metabolites were identified based on metabolite-specific fragmentation patterns in tandem-MS (MS/MS) (online Supplementary Table 1). LC was conducted with the 1290 Infinity Binary UPLC system (Agilent Technologies, Santa Clara, CA, USA). Tryptophan, 5-hydroxyindoleacetic acid, indole-3-acetic acid, indolelactic acid, IPA, indoxyl sulphate and kynurenic acid (KYNA) were detected with reverse phase (Zorbax Eclipse XDB-C18, particle size 1·8 µm, dimensions of 2·1 × 100 mm, Agilent Technologies, USA; injection volume of 1 μl) column, combined with orbitrap MS (Q Exactive Focus, Thermo Scientific, Bremen, Germany). Serotonin and kynurenine were detected with hydrophilic interaction chromatography (Acquity UPLC BEH Amide 1·7 µm, 2·1 × 100 mm, Waters, Ireland; injection volume of 3 μl) column, combined with quadrupole time-of-flight MS (6540 UHD Accurate-Mass Q-TOF MS, Agilent Technologies, Santa Clara, CA, USA). After both reverse phase and hydrophilic interaction chromatographic runs, both positive and negative jet stream electrospray ionisation modes were applied. Tryptophan and indoxyl sulphate were detected in negative ionisation mode, while others were detected in positive ionisation mode.

### Statistical analyses

Statistical justification and calculation of sample size have been described earlier^(23,24)^. Statistical analyses were performed using IBM SPSS Statistics software (IBM Statistics for Windows, version 27.0, USA). The normality of distribution of variables and residuals was assessed by Kolmogorov–Smirnov test. Spearman’s correlation was used to assess the association between metabolite abundances and GHQ scores, and metabolite abundances and the mean whole-grain intake during the WM.

In the whole study group, the association between GHQ scores (independent variable) and the mean whole-grain consumption (dependent variable) was assessed using linear regression analysis (unadjusted model/model 1). Similarly, the association between the mean whole-grain consumption (independent variable) and metabolite abundances (dependent variables) was assessed using linear regression analysis (unadjusted model/model 1). As for the GHQ scores and metabolites, the values obtained from the measurements conducted at the end of the WM were included in the analysis. Logarithmic (natural) transformation was made for measurements of the non-normally distributed metabolites in order to meet the assumptions of the regression analyses. The model with GHQ scores and the mean whole-grain consumption was further adjusted for confounding by including age, sex, body weight change during the WL and the mean energy intake during the WM as covariates (adjusted model/model 2). The analysis was not controlled with the intervention groups (higher-satiety food/lower-satiety food) as the intervention diets affected whole-grain intake. This could have provided an unnecessary adjustment and thus eliminated the effects of the whole-grain consumption. The models with the mean whole-grain consumption and tryptophan metabolites were further adjusted for confounding by including in the models age, sex, mean energy intake during the WM, metabolite abundance before the WM and either weight change during the WL (model 2) or metabolite abundance before the WL (model 3). Furthermore, a sensitivity analysis was conducted by adjusting for age, sex, metabolite abundance before the WM, metabolite abundance before the WL and the mean protein intake during the WM.

Lastly, to follow potential nonlinearity of the association, participants were divided into three GHQ groups by manually splitting the study population into tertiles based on the GHQ scores before the WL (the score ranges for the lowest, middle and highest tertiles are 3–8, 9–11 and 12–25, respectively). The cut-off points of the scores were set so that the group sizes were as even as possible in terms of the number of participants. Differences in tryptophan metabolite abundances between the GHQ groups were assessed with linear mixed model analysis with group, time, group × time interaction, sex and age as fixed effects and participant as a random effect. If the effect of group × time interaction was not statistically significant, it was removed from the models to investigate the effect of time on metabolite abundances. In case of a significant effect of group × time or time, *post hoc* pairwise comparisons of study weeks were calculated to assess between-group or within-group differences in metabolites at different timepoints. Logarithmic (natural) transformation was made for measurements of tryptophan metabolites to meet the model assumptions.

## Results

### Participants

Eventually, eighty-two subjects completed the whole weight management intervention study. Three participants were excluded from the current study due to the lack of metabolomic analyses. Therefore, seventy-nine participants were included in the regression analyses. Characteristics of the seventy-nine participants are shown in Table 2. In addition, one participant was excluded from the GHQ groups due to missing GHQ score from baseline before the WL. Therefore, seventy-eight participants were included in the linear mixed model analysis.

**Table 2.** Characteristics of the participants included in the current study (mean values and standard deviations)

	Mean	sd
*n*	79	
Females/males	58/21	
Age (years)	49·7	9·0
Body weight before the WL (kg)	95·4	12·0
BMI before the WL (kg/m^2^)	34·2	2·5
Weight change during the WL (kg)	–12·1	3·5
GHQ scores before the WL	10·9	4·6
Whole-grain consumption during the WM (g/d)	115·1	45·2

GHQ, General Health Questionnaire; WL, weight loss; WM, weight maintenance.

### Association between GHQ scores at the end of the weight maintenance and the mean whole-grain consumption during the weight maintenance

First, the whole study group was examined using linear regression models. Due to the absence of food diaries for some participants at week 24 of the WM, the mean whole-grain consumption during the entire WM was determined using the available food diaries. In this way, it was possible to retain the maximum number of participants in the sample. No significant association was found between the mean whole-grain consumption and GHQ scores (Table 3). Despite not reaching statistical significance, there was a potential association indicated by the 95 % confidence interval when considering the impact of age, gender and energy intake.

**Table 3.** Association between GHQ scores at week 24 of the weight maintenance period and mean whole-grain consumption during the weight maintenance period in the whole study sample (95 % confidence intervals)

	Mean whole-grain consumption during the WM			
	R^2^	B	95 % CI	*P*
GHQ scores at week 24 of the WM				
Unadjusted	0·006	–0·9	–3·3, 1·6	0·487
Adjusted*	0·236	–2·0	–4·3, 0·3	0·084

GHQ, General Health Questionnaire; WM, weight maintenance.

* The adjusted model of linear regression analysis was adjusted for age, sex, mean energy intake during the WM, and weight change during the WL.

### Association between the mean whole-grain consumption during the weight maintenance and tryptophan metabolites at the end of the weight maintenance

**Fig. 1.**
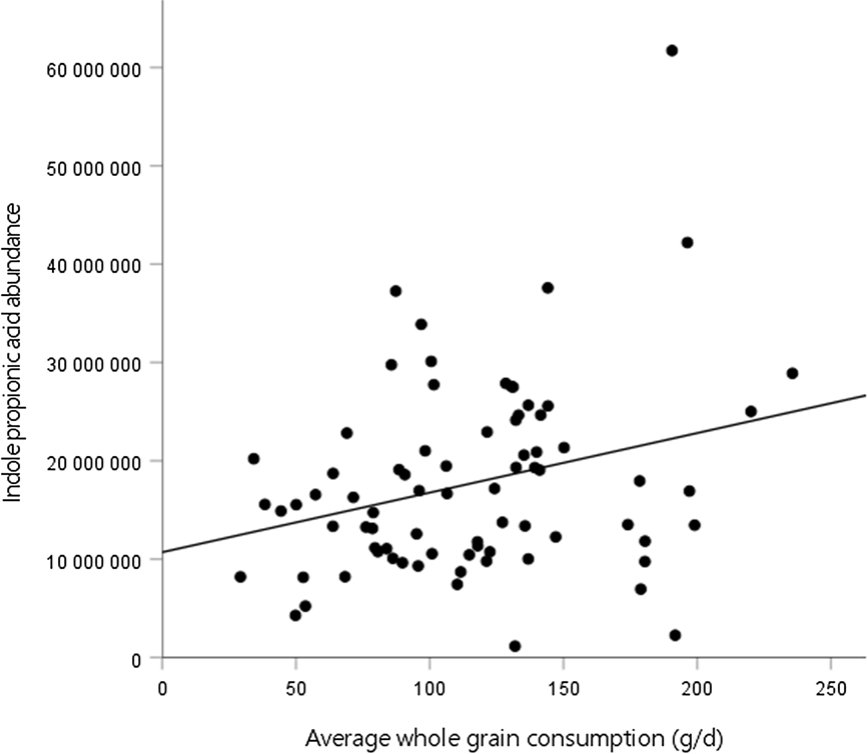
Association between whole-grain consumption during the weight maintenance (WM) and indole propionic acid abundance at week 24 of the WM.

Second, we assessed the association between the mean whole-grain consumption during the WM and tryptophan metabolites at week 24 of the WM. In model 3, there was a positive association between the mean whole-grain consumption and IPA abundance at the end of the WM (Fig. 1, Table 4). No significant associations were identified between the mean whole-grain consumption and any other metabolites. Furthermore, there were no significant associations between the mean whole-grain consumption and any metabolites in model 2 (online Supplementary Table 2).

**Table 4.** Association between the mean whole-grain consumption during the weight maintenance period and tryptophan and its metabolites at week 24 of the weight maintenance period in the whole study sample (95 % confidence intervals)

	R^2^	B	95 % CI	*P*
IPA				
Whole-grain consumption*	0·855	59 529·5	5008·5, 114 050·5	0·033
IAA				
Whole-grain consumption*	0·481	4662·6	–51435·5, 60 760·8	0·869
IS				
Whole-grain consumption*	0·393	126·3	–32871·4, 33 124·1	0·994
5-HIAA**				
Whole-grain consumption*	0·635	2·7 × 10^−5^	–0·0, 0·0	0·991
Tryptophan				
Whole-grain consumption*	0·423	–1912·4	–12502·9, 8678·2	0·720
ILA				
Whole-grain consumption*	0·548	1511·2	–8053·6, 11 076·0	0·754
KYNA				
Whole-grain consumption*	0·526	–616·3	–1413·8, 181·3	0·128
Serotonin				
Whole-grain consumption*	0·463	–1·742	–606·5, 603·0	0·995
Kynurenine**				
Whole-grain consumption*	0·365	0·00	–0·0, 0·0	0·750

IPA, indole propionic acid; IAA, indole-3-acetic acid; IS, indoxyl sulphate; 5-HIAA, 5-hydroxyindoleacetic acid; ILA, indolelactic acid; KYNA, kynurenic acid.

* Linear regression analysis was adjusted for age, sex, regression was adjusted with metabolite abundance before the WM, mean energy intake and metabolite abundance before the weight-loss period.

** Log(natural) transformed values were used in the analysis.

The overall protein intake was considered to potentially affect the tryptophan metabolism over the intervention. Thus, the sensitivity analysis using adjustment with mean protein intake during the WM was conducted. Interestingly, the negative association was observed between the mean whole-grain consumption and KYNA abundance at week 24 of the WM, differing from the energy-adjusted analysis. Simultaneously, the association between whole-grain consumption and IPA lost its statistical significance (online Supplementary Table 3).

### Tryptophan metabolism in GHQ groups

Finally, we examined the differences in metabolite abundances between participants divided into tertiles of GHQ. Descriptive information of the participants in the GHQ groups is represented in Table 1.

**Fig. 2.**
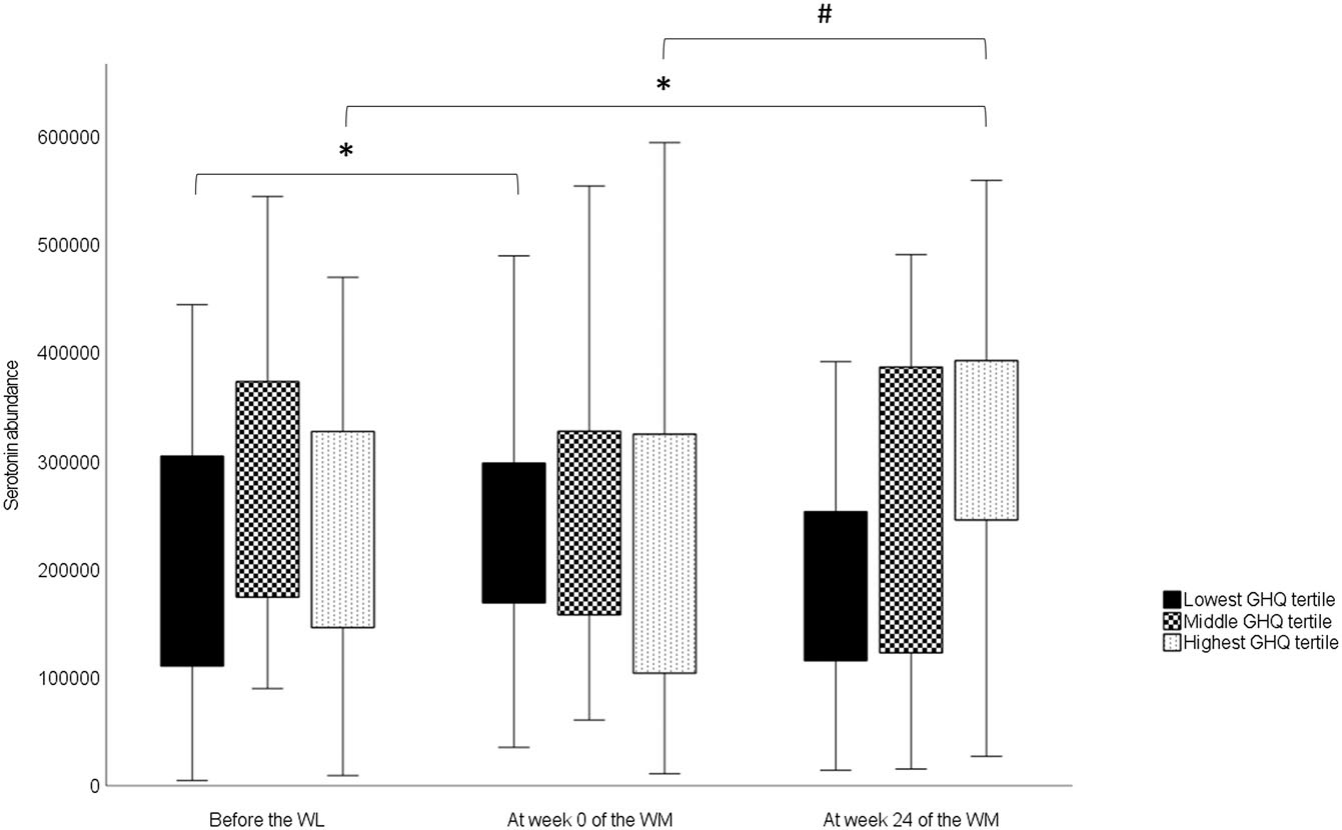
Changes in serotonin abundances in the GHQ tertile groups during the study obtained from a linear mixed model. In the box plots, the boundary of the box closest to zero indicates the 25th percentile, and the boundary of the box farthest from zero indicates the 75th percentile. Whiskers above and below the box indicate the minimum and maximum values. WL, weight loss; WM, weight maintenance; GHQ, General Health Questionnaire. *Significantly different from before the WL value (*P* < 0·05). ^#^Significantly different from the value of week 0 of the WM (*P* < 0·001).

For serotonin, there was a significant overall time × group interaction (Table 5). However, no significant differences were observed in serotonin levels among the GHQ tertiles at any timepoints according to *post hoc* analysis. In the lowest GHQ tertile, serotonin abundance at week 0 was significantly higher than the abundance before the WL (*P* 0·034) (Fig. 2). In the highest GHQ tertile, serotonin abundance at week 24 of the WM was significantly higher than the abundance before the WL (*P* 0·015) and week 0 (*P* 0·001). No significant effect of group × time interaction was found for the other metabolites.

**Table 5.** Differences in tryptophan metabolites in GHQ tertile groups at baseline, week 0 and week 24

	Group × time			Group			Time		
	F(df)		*P*	F(df)		*P*	F(df)		*P*
IPA*	1·013	4, 150·0	0·403	1·919	2, 73·0	0·154	18·324	2, 154·0	<0·001
IAA*	1·256	4, 150·0	0·290	0·689	2, 73·0	0·505	0·239	2, 154·0	0·788
IS*	1·246	4, 150·0	0·294	1·195	2, 73·0	0·309	22·470	2, 154·0	<0·001
5-HIAA*	1·167	4, 150·0	0·328	0·882	2, 73·0	0·418	19·424	2, 154·0	<0·001
Tryptophan*	1·082	4, 150·0	0·368	0·030	2, 73·0	0·971	49·926	2, 154·0	<0·001
ILA*	1·057	4, 150·0	0·380	0·727	2, 73·0	0·487	30·906	2, 154·0	<0·001
KYNA*	1·314	4, 150·0	0·267	0·704	2, 73·0	0·498	11·749	2, 154·0	<0·001
Serotonin*	4·235	4, 150·0	0·003	0·194	2, 73·0	0·824	0·813	2, 154·0	0·445
Kynurenine*	0·979	4, 150·0	0·421	0·942	2, 73·0	0·394	41·411	2, 154·0	<0·001

IPA, indole propionic acid; IAA, indole-3-acetic acid; IS, indoxyl sulphate; 5-HIAA, 5-hydroxyindoleacetic acid; ILA, indolelactic acid; KYNA, kynurenic acid.

* Log(natural) transformed values were used in the analysis.

When the group × time interaction was removed from the model, the effect of time was significant on every metabolite but indole-3-acetic acid and serotonin. The metabolite levels at week 0 of the WM were significantly higher than the levels before WL and week 24 of the WM for IPA and 5-hydroxyindoleacetic acid (*P* < 0·001). Meanwhile, the metabolite levels at week 0 of the WM were significantly lower than the levels before the WL and week 24 for indoxyl sulphate, tryptophan and indolelactic acid (*P* < 0·001). For KYNA and kynurenine, the levels before the WL were significantly higher than the levels of week 24 (*P* 0·017 and < 0·001, respectively), and the levels at week 0 were significantly lower than the levels before WL (*P* < 0·001) and week 24 (*P* 0·016 and < 0·001, respectively) in the groups.

## Discussion

In this study, we evaluated associations between whole-grain consumption, tryptophan metabolism and psychological distress in participants who took part in a weight management intervention study. To our best knowledge, this is the first study investigating these associations collectively. Specifically, there is still limited evidence of alterations in tryptophan metabolism among groups with varying levels of psychological distress. The mean whole-grain consumption during the WM was positively associated with the plasma levels of tryptophan metabolite IPA at the end of the WM. In addition, serotonin levels changed differently in the GHQ tertile groups. Serotonin levels were higher after the WL in the lowest GHQ tertile, while the level at the end of the WM was higher compared with other timepoints in the highest GHQ tertile. Levels of other metabolites changed similarly in the whole study group during the study. On the other hand, psychological distress was not associated with the mean whole-grain consumption during the WM.

IPA is an indole derivative produced by the gut microbiota. It has shown neuroprotective properties in mice by suppressing immune responses and central nervous system inflammation^(29)^. In addition, higher levels of IPA have been associated with decreased risk of type 2 diabetes^(30)^, whereas lower levels were associated with cardiovascular events^(31)^ and liver fibrosis^(32)^. Our finding of a positive association between IPA and whole-grain consumption is consistent with previous studies, as IPA has been related to whole-grain consumption in a cohort study^(33)^, in a subgroup of participants of a randomised controlled study^(12)^ and a subgroup of men of a prospective cohort study^(34)^. This association may be explained by the effects of whole grains on gut microbiota. Whole grains are rich in dietary fibre, which influences the composition and metabolism of gut microbiota^(10)^. In fact, dietary fibre intake has been related to IPA-associated bacterial genera in human faeces^(33)^, and interaction between IPA-producing species and dietary tryptophan intake on plasma IPA concentrations was apparent among men with higher dietary fibre intake^(34)^. Therefore, our results provide further insight into the link between whole-grain intake and IPA production in the gut. Moreover, the results prompt the inquiry of whether the indole pathway could serve as one of the connections between the consumption of whole grains and their potential health benefits.

The further sensitivity analysis was done to apply adjustment with mean protein intake. This analysis revealed the negative association between KYNA and whole-grain intake. Similarly, a negative association between the whole-grain consumption and KYNA levels was observed in a previous study^(33)^. Increasing protein intake also increases overall amino acid intake, potentially leading to competition in epithelial transport, as is known to happen between other neutral amino acids and tryptophan^(35)^. This could result in alterations in tryptophan metabolism, as more tryptophan reaches the colon, where gut microbiota may utilise it in tryptophan metabolism, potentially contributing to the indole pathways. Nevertheless, in the sensitivity analysis, the association between IPA and whole-grain intake was no longer significant. Thus, taking into account the variation in the food consumption in the study population targeted to fibre and protein intake, we cannot rule out the potential effects of overall protein intake on the findings in tryptophan metabolism.

Contrary to our findings, results of other studies have indicated that whole-grain consumption is related to also tryptophan and other metabolites than IPA or KYNA. After a 4-week intervention period including whole grains, plasma tryptophan levels were lower compared with a period of refined grain consumption in healthy adults^(36)^. However, the same was not observed in another study comparing 8-week rye bread and white wheat bread interventions in menopausal women^(37)^. For serotonin, higher whole-grain intake has been associated with either lower^(11)^ or higher plasma levels^(33)^. Other indole derivatives than IPA have been associated with the consumption of whole-grain products in a cohort study^(33)^ and after a rye bread intervention^(37)^. In addition, an inverse association between whole-grain consumption and kynurenine and its derivatives has been found^(33)^. However, it is important to acknowledge that the diversity of the participants in the previous studies and the present study makes it challenging to establish a comprehensive perspective on the relationships. Unlike previous studies, our study included participants who had obesity and underwent WL intervention. Weight change and its effects on tryptophan metabolism may have affected the assessment of association between whole-grain consumption and tryptophan metabolism^(15–17)^. Adults with overweight or obesity have an altered tryptophan metabolism compared with people with normal body weight, with plasma kynurenine levels being elevated and tryptophan and indole derivate levels being decreased^(13,14)^. This has not, however, been observed in all studies^(38)^. Furthermore, in adults with overweight, WL with low-calorie diets resulted in decreased serum levels of indole^(15)^, tryptophan and kynurenine^(16,17)^. The extensive WL achieved during the current study may have influenced tryptophan metabolism even during the WM, potentially confounding the detection of associations between whole-grain consumption and metabolites. To address this prolonged effect of WL, the statistical models were adjusted for the WL outcomes during the WL.

The lack of observed associations between whole-grain consumption and metabolites in the present study may be due to small sample size and great variations in metabolite abundances between the participants as well. Furthermore, despite the intervention, the average consumption of whole grains was not necessarily significant enough to induce notable changes in metabolite levels. Therefore, further research is needed to clarify the mechanisms underlying the association between whole-grain intake and tryptophan metabolism and to determine the level of whole-grain consumption that benefits tryptophan metabolism.

One factor that influences the availability of tryptophan and, consequently, its metabolism is the dietary intake of tryptophan. As tryptophan is an amino acid, the intake of protein inherently affects the intake of tryptophan. Among whole grains, tryptophan is present in protein-rich foods, including milk, poultry meat, cheese and nuts^(8)^. Increased dietary protein intake can result in elevated levels of free tryptophan in the plasma^(39)^, and a higher intake of dietary protein has been shown to impact both endogenous and colonic microbial tryptophan metabolisms in humans^(40–43)^. In our study, participants initially underwent the WL with modified dietary protein intake, after which the protein sources were reintroduced into their diet. Additionally, intervention groups consumed products with different amounts of protein during the WM. This probably resulted in alterations to the intake of dietary tryptophan as well. Indeed, within the sensitivity analysis adjusted for the mean protein intake, a positive association between the mean whole-grain consumption and KYNA at the end of the WM was revealed. However, adjusting with mean energy intake was considered to account for variations in overall food consumption, caloric and nutrient intake, including whole grains and both protein- and tryptophan-rich foods. Additionally, diverse energy requirements among individuals may lead to distinct dietary patterns, influencing tryptophan metabolism and subsequent production of tryptophan metabolites. Hence, incorporating mean energy intake may improve result precision, and considering both energy and protein intake in the analysis would pose concerns related to multicollinearity.

We also found that serotonin levels increased during the WL in the group with the lowest pre-WL GHQ scores and during the WM in the group with the highest pre-WL GHQ scores. Previously, plasma serotonin levels have correlated negatively with depression and anxiety in patients with chronic kidney disease^(44)^ and type 2 diabetes^(45)^. In our study, GHQ scores declined during the WL and then slowly returned towards the baseline level in the whole study group^(23)^. Therefore, our findings are consistent with previous research on the association between psychological well-being and plasma serotonin levels in the group with the lowest GHQ scores. However, this association was not observed in the highest GHQ tertile group. It is noteworthy that in prior studies^(44,45)^, the participants had major metabolic disorders that may have influenced the results. Consequently, caution must be exercised when comparing these findings with those of healthy or obese individuals like the ones in our study.

In the brain, serotonin participates in the regulation of emotions, cognition and food intake, although the exact mechanisms behind these processes are not yet fully understood^(18–20)^. Nonetheless, serotonin levels in our study reflect most probably changes in dietary intake during the study, since plasma serotonin levels are thought to reflect gut-originated serotonin^(46)^. Peripheral serotonin cannot cross the blood–brain barrier, and thus, it has separate biological functions from the serotonin produced in the brain^(47–49)^. However, gut-originated serotonin can affect the central nervous system via the gut–brain axis, namely, via vagal signalling from the gut to the brain^(50,51)^. Stimulation of the vagus nerve by peripheral serotonin has been observed to enhance the activity of serotonergic neurons, consequently influencing serotonin concentrations in the brains of rats^(50)^. In addition, after surgically blocking vagal gut–brain communication, selective serotonin reuptake inhibitors lose their antidepressive effects in mice^(51)^. These findings suggest a potential link between the gut, serotonin and psychological well-being and highlight the importance of further investigation of the role of the gut–brain axis in the regulation of mood and behaviour.

Given the association between whole-grain consumption and serotonin metabolism in prior studies^(11,33,44)^, it is reasonable to investigate this perspective here. Whole-grain consumption changed significantly during the study; during the WL, whole grains were completely excluded from the participants’ diets, while consumption during the WM was partially determined according to the study design. Nonetheless, there was no difference in whole-grain consumption between GHQ tertile groups during the WM, indicating that it is not the reason for the varied responses observed between the lowest and highest GHQ tertiles. It is possible that there are differences in unmeasured factors that may influence serotonin levels in the peripheral circulation between the tertiles. For example, the intake of nutritional factors that affect the release of serotonin in the gut, such as glucose^(52)^, may have differed quantitatively among the tertiles. Thus, the factors underlying the different serotonin responses remain unclear.

In addition to serotonin, other metabolites have previously been associated with psychological well-being^(44,53,54)^. Plasma IAA was correlated with anxiety and depression in patients with chronic kidney disease^(44)^, while higher kynurenine levels were related to a lower severity of depressive symptoms in adults with depression^(53)^. Furthermore, compared with adults or elderly populations without depressive symptoms, plasma tryptophan was lower and kynurenine, KYNA and quinolinic acid were higher in elderly individuals with mild or moderate depressive symptoms^(54)^. However, we found no differences in tryptophan metabolites between the GHQ groups but only changes in their levels within the groups during the study. As discussed earlier, alterations in body weight and dietary intake during the study might have affected tryptophan metabolism. Participants underwent WL period with very-low-energy diet, after which they followed one of the intervention diets through the WM. Consequently, the diets differed significantly between the two periods. Levels of several metabolites altered during the WL, only IAA being an exception. Kynurenine and tryptophan levels decreased during the WL, confirming previous findings^(17)^. In addition, there were great variations in metabolite abundances which may partly explain our results. The variance in the study designs between our research and previous studies might account for the differing results as well.

Lastly, we did not find an association between whole-grain consumption and psychological distress. Similarly, in a previous study, there were no differences in whole-grain intake between depressed and non-depressed participants^(55)^. However, in our earlier study, the consumption of whole-grain products was lower in the most stressed individuals compared with the least stressed^(6)^. Additionally, López-Ceperon *et al.* ^(7)^ reported that psychological distress was connected to a lower intake of fibre-rich foods, including whole grains. It is essential to note that the effect size of our analysis on whole-grain consumption and GHQ scores was small, which may partly explain the lack of significance in our sampling. In other words, had the outcome been statistically significant, it would have been challenging to assert that individuals with higher psychological distress significantly consumed fewer whole-grain products compared with those with lower stress levels. It is also possible that the sample size in this study was insufficient for the effect size to attain statistical significance.

This study has certain strengths. The chosen methods enabled the inclusion of nearly all participants from the original study in the present analysis, maximising the sample size. The study design incorporates the variability in whole-grain consumption within the study population. Taking multiple measurements during the study helps to even out day-to-day variation, providing a more accurate representation of participants’ whole-grain consumption. In addition, viewing consumption as mean consumption during the WM ensured sufficient size of the study sample in analyses. Nonetheless, there were limitations as well. The basis for defining whole grains in this study relied on the fibre content rather than the proportion of whole grains in cereal foods. The database that was used in the current study enabled the categorisation of the products on the market at the time of the study based on their fibre content. In addition, the assessment of whole-grain consumption was based on self-reported food diaries, which may involve misreporting. In addition, the intervention foods covered 30 % of daily energy intake during the WM^(23)^. Thus, the participants could not eat entirely *ad libitum* as they were instructed to consume the recommended number of either whole-grain bread or bread with less fibre. This may have interfered with the analysis of the relationship between psychological distress and whole-grain consumption. Nonetheless, as stated earlier, an overadjustment may have occurred if we had controlled the analysis with the intervention groups, and thus, the intervention groups were left out from the analyses. Secondly, the food diaries, GHQ and metabolites were not collected at the same timepoints during the WM. Hence, we could not take longitudinal perspective to our analysis, and more longitudinal studies are needed to clarify the causality between whole-grain consumption, tryptophan metabolism and psychological distress. It is evident that there might be several other factors that may contribute to the associations shown in the present study, especially due to the multidimensional phenomena, such as psychological well-being, tryptophan metabolism and food consumption, studied in the present study. Thus, we need to be careful when making interpretations based on the present findings.

In conclusion, higher whole-grain consumption was associated with higher levels of IPA, which is possibly explained by the effects of dietary fibre on the gut microbiota. Tryptophan metabolism changed during the study in the whole study group, independently from the level of psychological distress. However, we cannot rule out the possibility that the overall protein intake may have an impact on tryptophan metabolism during the study. In addition, whole-grain intake and psychological distress were not associated with each other in this study population. Therefore, this study suggests the potential favourable effect of whole-grain consumption on the indole pathway, particularly through the production of IPA.

## Supporting information

Liikonen et al. supplementary material 1 Liikonen et al. supplementary material

Liikonen et al. supplementary material 2 Liikonen et al. supplementary material

Liikonen et al. supplementary material 3 Liikonen et al. supplementary material

## Supplementary material

For supplementary material/s referred to in this article, please visit https://doi.org/10.1017/S0007114524001077
